# Adjuvant Treatment of Melanoma

**DOI:** 10.1155/2013/545631

**Published:** 2013-02-17

**Authors:** J. A. Moreno Nogueira, M. Valero Arbizu, R. Pérez Temprano

**Affiliations:** ^1^Department of Oncology, Virgen del Rocio University Hospital, Royal Academy of Medicine, 41001 Seville, Spain; ^2^Department of Oncology, Infanta Luisa Hospital, 41010 Seville, Spain; ^3^Department of Medicine, Virgen Macarena University Hospital, 41014 Seville, Spain

## Abstract

Melanomas represent 4% of all malignant tumors of the skin, yet account for 80% of deaths from skin cancer.While in the early stages patients can be successfully treated with surgical resection, metastatic melanoma prognosis is dismal. Several oncogenes have been identified in melanoma as BRAF, NRAS, c-Kit, and GNA11 GNAQ, each capable of activating MAPK pathway that increases cell proliferation and promotes angiogenesis, although NRAS and c-Kit also activate PI3 kinase pathway, including being more commonly BRAF activated oncogene. The treatment of choice for localised primary cutaneous melanoma is surgery plus lymphadenectomy if regional lymph nodes are involved. The justification for treatment in addition to surgery is based on the poor prognosis for high risk melanomas with a relapse index of 50–80%. Patients included in the high risk group should be assessed for adjuvant treatment with high doses of Interferon-**α**2b, as it is the only treatment shown to significantly improve disease free and possibly global survival. In the future we will have to analyze all these therapeutic possibilities on specific targets, probably associated with chemotherapy and/or interferon in the adjuvant treatment, if we want to change the natural history of melanomas.

## 1. Introduction

Melanomas constitute 2-3% of all cancers, 95% presented as skin cancer and only 5% in nonskin locations. It represents 4% of all malignant tumors of the skin, yet account for 80% of deaths from skin cancer. The incidence in Spain adjusted per 100,000 populations is 5.85 for men and 7.50 for women, according to published data. In the U.S. 76,250 new cases are expected for 2012 (44,250 men and 32,000 women) and a mortality rate of 9180 patients. Using data from 2005–2009 the age-adjusted incidence was 21.0 per 100,000 (27.2 for men and 16.7 for women). The highest incidence occurs in Australia and New Zealand (38/100.000 inhabitants) and Japan has just 0.47 per 100,000 population, which is indicative of the different incidence rates by geographic areas.

Globocan's data from 2008 shows an incidence of 199.627 cases and a mortality of 46.372 (M: 101.807/25.860 and W: 97.820/20512), when in 2002 the incident was 160.000 (M : F sex ratio, 0.97) and the mortality 41.000 (M : F sex ratio, 1.2) [1]. Melanoma is one of the neoplasms that is increasing the most, both young and old, exceeded only by the liver and thyroid cancer. It is currently the fifth most common cancer in men and the seventh in women. While in the early stages patients can be successfully treated with surgical resection, metastatic melanoma prognosis is dismal, with a mortality rate of 90% in five years.

The majority of melanomas originate in existing nevi; only 30% are new lesions. Radial or spreading growth initially appears (malignant lentigo melanoma, superficial acral lentiginous melanoma) followed by vertical growth that involves lymphatic colonisation. Nodular melanoma only presents vertical growth, without any previous radial growth phase and this is why it has a worse prognosis. The Clark levels of invasion (I, II, III, IV, and V) and Breslow's tumour thickness measurements (0.5, 1.0, 1.5, 2.0, 2.5, 3.0, 4.0, and 5.0 mm) are based on the growth depth of histopathological studies and enable evaluating the prognosis and estimating the risk after surgery of the primary tumour [2]. They indicate the risk of metastasis and are the bases and foundations of studies of tumour extension and classification. 

One important step is the study of the sentinel lymph node, which enables precise classification of lymph node affectations. This has good prognosis value and influences making later therapeutic decisions such as the use of high dose adjuvant interferon. Furthermore, in cases where these nodes are positive, it indicates the advantages of early lymphadenectomy. Its indication is for stages I-II of the AJCC, which is without evidence of regional or distant lymph node metastases that may include ultrasonography [3]. The Breslow degree of millimetric invasion informs about the risk of hidden metastasis at a lymph node and distal level. If this degree of invasion is less than 1 mm, the positivity of the sentinel lymph node is only 0–5% and the cure rates by surgery are 98%. This means that in this group the indication of performing the sentinel lymph node technique is not logical because of its low indicative value. In patients with a degree of invasion between 1 and 4 mm (T2, T3) the positivity ranges are from 1 to 14% in T2 and from 11 to 34% in T3. This is why the sentinel lymph node technique would be very important in this group because of its prognostic and therapeutic repercussions.

In patients with a degree of invasion of more than 4 mm (T4), the risk of regional micrometastasis is very high, between 20–65%, as is that of distant micrometastasis (>60%). This means that the sentinel lymph node technique would be less informative regarding palliative lymphadenectomy and the indication of treatment with high dose interferon. This is because this procedure would be indicated from the outset, as the patients are in high risk. Nevertheless, it would enable adequate classification and this would be valuable when planning future clinical trials with more homogeneous groups of patients (Table 1) [4].

A histopathological study of the primary lesion and complementary examinations are the basis of the stage classification as a step prior to planning therapy following surgery. The pathological examination of the primary lesion and complementary studies will help us staging the disease in order to establish a treatment planning. Clark's invasion degree and Breslow's thickness measure vertical growth melanoma (“T”) while additional examinations will appreciate regional nodal involvement (“N”) and distant metastases (“M”). The 2010 classification of the AJCC/UICC (seventh edition) has evident differences compared to that of 1997. It simplifies the Breslow scale to 1, 2, and 4 mm and considers the presence or not of ulceration and also assesses the number of mitosis/mm^2^. It adequately classifies lymph node affectation and, in the metastatic phase, distinguishes types of metastasis and the prognosis value of high LDH levels. This disease classification finally includes assessment of the sentinel lymph node. All this enables identification of the different stages as well as different risk groups, an important aspect for deciding complementary treatments [5, 6]. Future studies of gene expression profiles by microarray techniques will complete the diagnosis of melanoma and may properly outline susceptibility, prognosis, and treatment individualization.

Patients with stages I and II have no distant lymph node metastases and survival rates of 40% to 95%, as defined by the degree of infiltration and the presence or not of ulceration. This means that substage IIA is only considered of intermediate risk when it is ulcerated (Breslow 1.1–2 mm) or has a thickness of 2.1–4 mm without ulceration. High risk patients include substages IIB (Breslow 2.1–4 mm ulcerated or >4 mm nonulcerated) and IIC (Breslow > 4 mm ulcerated). The variability of survival in these stages indicates its heterogeneity, and so other prognostic factors (mitotic rate, serum YKL-40, PTEN, Ki67 expression, etc.) must be included to better discriminate different patient subgroups [7–10]. Stage III patients are also a very heterogeneous group, with high risk and worse prognosis as they always involve lymph node affectation where the number of affected nodes is an indicator of survival, with age, location, the presence of macro- or micrometastasis in the lymphatic nodes (67% versus 43% up to 5 years, *P* < 0.001), and so forth also having an influence (Tables 2 and 3) [11, 12]. In this stage and in the near future, other factors must be considered such as serum protein S-100B levels, which are an independent prognostic factor as an initial baseline measurement, and also, during the followup, different gene expressions, circulating melanoma cells, and so forth as they would provide information in addition to standard clinical and histological information, bring about an improvement in the precision of both the diagnosis and the prognosis, and contribute, as already mentioned, to this therapeutic future [13–18].

The strong relationship between gene mutations and the location of the primary melanoma (Table 4) is now known. These mutations lead to activation of the MAPK pathway that increases cell proliferation, prevent apoptosis, and promote angiogenesis. Several oncogenes have been identified in melanoma as BRAF, NRAS, c-Kit, and GNA11 GNAQ, each capable of activating MAPK pathway, although NRAS and c-Kit also activate PI3 kinase pathway, including being more commonly BRAF activated oncogene. 80 to 90% of BRAF mutations are due to a substitution of glutamate for valine at position 600 in the amino acid sequence of BRAF. The second most common mutation is a substitution of the lysine in the same position. The two substitutions (V600E and V600K) represent 95% of all BRAF mutations in melanoma, and both create a constitutively active kinase that is independent of receptor tyrosine kinase or RAS. As a serine-threonine kinase, which consumes ATP as an energy source, BRAF makes it a target for ATP competitive inhibitors such as small molecular; being the most developed, vemurafenib, dabrafenib, and trametinib in the future may play an important role in the adjuvant setting, either alone or associated with chemotherapy or interferon. Mutations in BRAF, c-KIT, and the ANR may be found in approximately 70% of all melanoma. These mutations are rarely simultaneously in the same tumor in similar proportions. The distribution varies depending on the mutation site of origin and also by the absence or presence of chronic sun damage. On the other hand there is evidence to suggest that BRAF mutations pose a greater risk of recurrence and death [19].

## 2. Adjuvant Treatment

The treatment of choice for localised primary cutaneous melanoma (stages I, II, and III) is surgery and if there is regional affectation of the lymph nodes or if the sentinel lymph node is positive, this must be completed with lymphadenectomy. The resection should be deep in accordance with the thickness of the primary lesion. The recommended width of the margins should be 1 cm, for lesions 1 mm thick. In melanomas of 1–4 mm, about 2 cm is recommended and for lesions of more than 4 mm, about 3 cm. Elective regional lymphadenectomy is not recommended unless it is established in the study of the sentinel lymph node and this is positive, as in up to 37% of these cases there are affected nodes. Therapeutic lymphadenectomy should be performed when lymph node metastases have been clinically diagnosed. Surgery should be assessed once again for the metastatic disease, especially for metastases of the skin or those attached to organs, as they may be candidates for adjuvant treatment.

The justification for treatment in addition to surgery is based on the poor prognosis for high risk melanomas with a relapse index of 50–80% and low five-year survival rates of around 25–70%. Another reason would be that the metastatic disease has no efficient treatment capable of significantly prolonging patient survival.

Patients included in the high risk group should be assessed for adjuvant treatment with high doses of interferon-*α*2b, as it is the only treatment shown to significantly improve disease-free and possibly global survival.

Different types of adjuvant treatment have been investigated and others are under study and pending results.

### 2.1. Adjuvant Treatment with Chemotherapy

In randomized studies, adjuvant chemotherapy has not shown any significant benefits, even at high doses with the support of autologous bone marrow (Table 5) [20].

### 2.2. Adjuvant Treatment with Biochemotherapy

Various studies, with contradictory results, have been published of combined treatment with chemotherapy and cytokines; nevertheless, this is an interesting line for further investigation. A first study with 138 patients, 71 treated with biochemotherapy (cisplatin + vinblastine + DTIC + IFN + IL2) compared to two treatments with high dose interferon-*α*2b, (33 patients) versus intermediate doses (33 patients), did not show any significant differences in the groups regarding GS and RFS [21]. A second study compared two cycles of DTIC 850 mg/m^2^ followed by interferon-*α*2b 3 mill./3 s.c., during six months, compared to observation in patients with stages IIa, IIb, IIIa, and IIIb. There were no significant differences regarding RFS and GS in low risk patients (IIa), but the differences were significant in high risk patients with an RFS at 5 years of 42% versus 17% (*P* = 0.0018) and a GS at 7 years of 51% versus 30% (*P* = 0.0077). The benefits were more evident in metastasis-free survival and the procedure has an acceptable toxicity [22]. On the other hand, a wide phase III study from DeCOG (*Dermatologic Cooperative Oncology Group*) with 441 patients with regional lymphatic clearance after having positive nodes compared IFN *α*2a and 3 MU s.c. three times a week (A), (A) plus DTIC 850 mg/m^2^ every 4–8 weeks for two years (B) and just observation (C). The results showed significant improvement in RFS and OS in group A versus C, but with no differences between B and C, meaning possibly that DTIC reverts the benefits of adjuvant IFN [23]. Recently, the study of the Intergroup S-0008 has compared high dose IFN versus biochemotherapy classical scheme (DTIC + cisplatin + vinblastine + IL2 + IFN with G-CSF) in patients at high risk (IIIA-N2a/IIIC-N3). 402 patients were included between 8/2000 and 11/2007, and in the current analysis, there are significant differences in RFS for the biochemotherapy without differences in OS, with greater grade IV toxicity. Therefore, biochemotherapy is a valid alternative for adjuvant treatment in patients with high risk melanomas [24].

There are also some studies of neoadjuvant treatment with biochemotherapy. One in stage III with 48 patients analyzed for the association of cisplatin + vinblastine + DTIC + IFL + IL2. At five years the GS was 66% and the RFS was 56%, higher than historic controls [25].

### 2.3. Adjuvant Treatment with Immunostimulants

Seven studies with nonspecific immunostimulants did not show any significant benefits (Table 6) [20].

### 2.4. Adjuvant Treatment with Vaccines

Various vaccines against melanoma are currently under development; some of them are in phase I, II, and III clinical trials, but in general they have not offered any advantages except in one study which only included 38 patients (Table 7) [20].

Melacine, a vaccination made from cell lysate, was compared to observation by SWOG (*Southwest Oncology Group*) in patients with melanoma of 1.5–4 mm in thickness without lymph node affectation. No benefit was observed but a retrospective analysis showed that the vaccinated patients that had positive HLA-A2 or C3 presented a disease-free survival of 77% compared to 64% of patients subjected to observation [27]. In the ECOG 1694 study, the group that received the vaccination of ganglioside GM2 activator protein fared worse than the group with high doses of interferon-*α*2b after a relatively short median followup. However this study did not include a control group without adjuvant treatment, and so it is not possible to determine whether vaccination with ganglioside was equivalent to observation or even prejudicial [28]. A recently presented randomized trial, where 604 patients in stage III were enrolled between April 1997 and January 2003, studied vaccination of allogeneic melanoma lysates with low doses of interferon-*α*2b, compared to high doses of interferon-*α*2b. At five years there were no differences in GS (61% versus 57%) or RFS (50% versus 48%) between both groups, but these figures were better than those for patients who did not receive any adjuvant treatment. The incidence of important side effects was similar, but the neuropsychiatric toxicity was higher in the second group [29]. The final results of the phase III study form EORTC 18961 have been presented in the ASCO 2010 meeting. The study had 1314 patients in stage II (T3-T4N0M0) recruited between March 2002 and December 2005. They were randomized after surgery to received GM2-KLH/QS-21 vaccination or to observation alone.The study had to be stopped because it did not show good results and the vaccination could be potentially harmful to patients (Table 8) [26].

### 2.5. Adjuvant Treatment with Interferon

At present the most common adjuvant treatment in high risk melanomas is interferon-*α*2b at high doses according to the Kirkwood scheme (induction: interferon-*α*2b: 20 million/m^2^, i.v., 5 days a week for four weeks; maintenance: interferon-*α*2b: 10 million/m^2^, s.c., three times a week for 48 weeks), which should also be assessed after surgery of the metastasis, without evidence of tumour.

Interferon is a glycoprotein described in 1957 by A. Isaacs and J. Lindemann as a product of virus infected cells that interfered with the replication of live virus in cell cultures. In the eighties, cloning by genetic engineering of a human interferon gene in *Escherichia coli* enabled the production of large amounts of interferon thus simplifying clinical research into cancer treatments. There are more than 20 varieties, but the three most important are interferon *α*, *β*, and *γ*, all being used in clinical oncology, especially *α* [30].

The genes that code interferon *α* and *β* are found in chromosome 9, whereas the gene coding the *γ* is in chromosome 12. Both *α* and *β* are structurally similar, with the same number of amino acids, the homology of the sequence of nucleotides being 45% and 29% for amino acids.

Interferon acts by binding to a specific membrane receptor protein, thus unleashing a cascade of signals whose end result is the expression of a certain number of genes. Interferon *α* and *β* share the same receptor, but *β* has greater affinity, with the gene of this receptor being found in chromosome 21 and that of interferon *γ* in chromosome 6 [31, 32].

The proteins produced as a result of gene activation and expression participate in different biological activities such as antiviral and immunomodulating actions, reduction of cell proliferation, suppression of gene expressions, inhibition of angiogenesis, induction of cell differentiation, and so forth. 

Oncological pathology essentially uses interferon *α* (IFN*α*-2a and IFN*α*-2b) as single agents or in combination with chemotherapy or other cytokines and monoclonal antibodies. Interferon-*α*2b was the first to be produced using the DNA recombinant technique and approved by the United States FDA. Over the last 15 years numerous studies have been carried out in various neoplasias, including lymphomas, CML, melanomas, and kidney cancer.

The antineoplastic activity of interferon has a double mechanism, it inhibits the proliferation and growth of tumour cells, directly affecting all phases of the cell cycle (M, G1, and G2), prolonging the cell cycle and reducing the number of cells that enter phases S and G2. The accumulative effect of prolongation of the cell cycle has cytostatic action and increases apoptosis. In second place it acts indirectly by inducing an increase of the antigen expression of the class I and II major histocompatibility complex on the surface of tumour cells, exercising an effect on modulation of the immune response to these cells. These antigens play an important role in recognition of neoplastic cells by cytotoxic T-cells together with increasing the effectiveness of all effector immune cells with cytotoxic capacity (NK cells, macrophages, etc.) on these tumour cells. The increased interferon induced expression of TNF-*α* receptors on the surface of these cells increases the cytostatic and cytotoxic action of TNF-*α* whose production is also increased. Something similar also occurs with other cytokines (CSF, IL1, etc.) that are involved in immune antitumorigenic cytotoxicity mechanisms [34, 35].

Another effect of the antitumour interferon is inhibition of tumour angiogenesis. Systemic treatment with interferon *α* and *β* reduces growth of endothelial cells that are essential for the formation of new vessels, by inhibition of angiogenic factors, thus having an indirect antiproliferation effect. Interferon *α* reduces the expression of FGF-2 and the transcription of VEGF. This is further enhanced by another possible mechanism, inhibition of IL-8, which has neoangiogenic capacity in numerous neoplasias [36].

Interferon has been widely investigated in melanoma, either as adjuvant treatment as well as in the metastatic setting.

**Figure 1:**
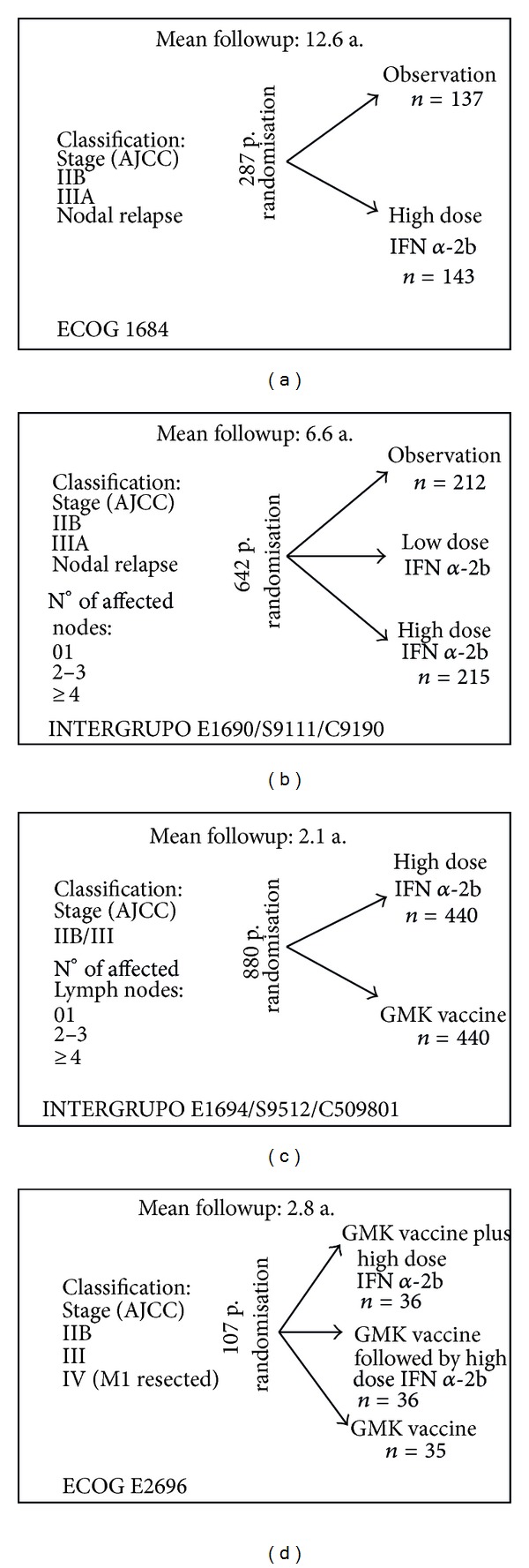
Eastern Cooperative Oncology Group and Intergroup. IFN [33].

The adjuvant treatment most recognised at present in high risk melanomas specially in USA is interferon-*α*2b at high doses and according to the Kirkwood scheme. This scheme has been used by the *Eastern Cooperative Oncology Group and Intergroup* to perform four randomized studies on 1916 patients whose data was updated in 2004 (Figure 1). The first study, E1684, showed that patients who received adjuvant treatment presented a recurrence-free survival (RFS) at five years of 37% compared to 26% (*P* = 0.0023) in the untreated group. The overall survival at five years was also significantly better (46% versus 37%, *P* = 0.0237) and this information enabled approval of IFN-*α* 2b as adjuvant treatment in high risk melanomas by the United States FDA, as well as the Spanish Ministry of Health. 

When this study was updated with a median followup of 12.6 years, it maintained the benefits in RFS (HR = 1.38, *P* = 0.02). The benefits for OS descended slightly (HR = 1.22, *P* = 0.18), but this could be due to deaths by other causes in the elderly population of the study (current mean age of >60 years).

The second E1690 study also showed benefits in RFS after a followup of 6.6 years (HR = 1.24; *P*_2_ = 0.09), but not so for OS.

**Figure 2:**
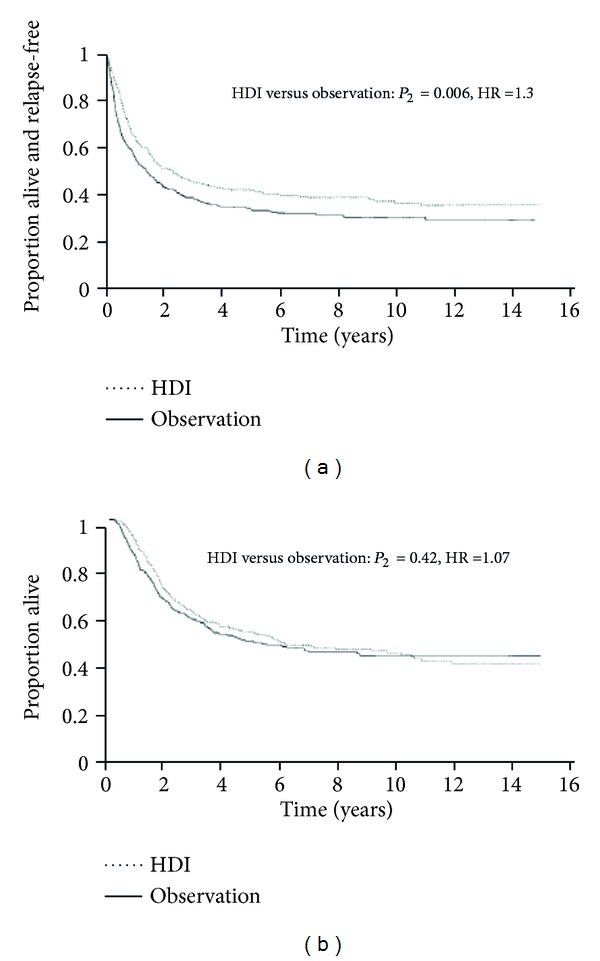
Joint analysis of the E1683 and E1690 studies recurrence-free survival/overall survival [33]. Proven benefits in relapse-free survival (HR: 1.30; *P*_2_ = 0.006), but not in overall survival.

In the combined analysis of these two ECOG studies with 713 patients and a median followup of 7.2 years, high doses of interferon-*α*2b were better than the observation group in regard to the RFS (HR = 1.30, *P* < 0.002). However this analysis showed no benefit in overall survival (OS) (HR= 1.08, *P* = 0.42) (Figure 2).

**Figure 3:**
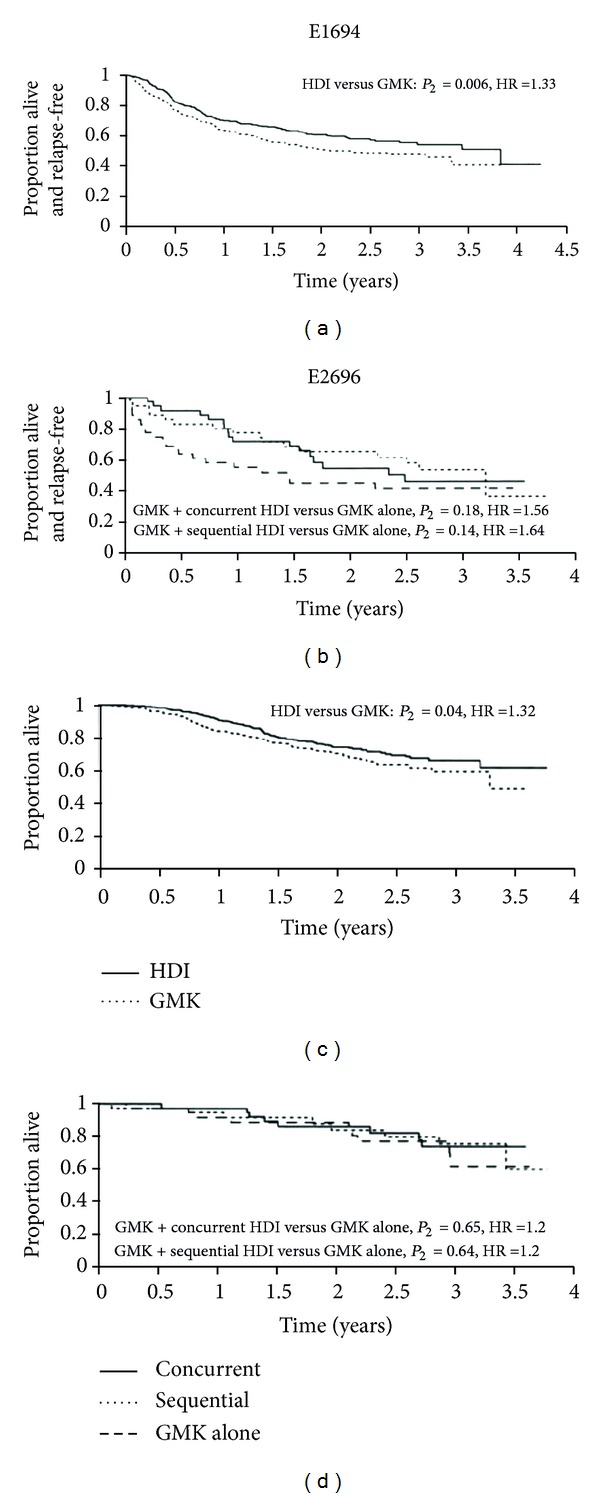
Studies E1694 and E2696. Recurrence-free survival/overall survival [33]. Study E1694 after a median followup of 2.1 years of high dose IFN*α*-2b shows better results compared to GMK vaccine for relapse-free survival (HR: 1.33; *P*_2_ = 0.006) and overall survival (HR: 1.32; *P*_2_ = 0.04). Study E2696 benefits from the combination of high dose IFN*α*-2b and GMK vaccine regarding relapse-free survival.

Study E1694 showed benefits in RFS and GS compared to GMK vaccine after a median followup of 2.1 years. Equally, study E2696 showed that the combination of GMK vaccine and interferon-*α*2b at high doses reduced the risk of relapse compared to GMK alone (Figure 3) [33].

In view of the above, it is possible to say that in patients with resected high risk melanoma interferon-*α*2b at high doses is an adjuvant treatment with clear evidence of increased RFS and moderate, but not significant, improvement of GS, with a toxicity that should be well assessed and explained to each patient, so that he/she participates in the decision making process. Adequate experience in the use of high dose interferon, with control of its toxicity and recommending good hydration, means that the majority of patients comply with the therapeutic plan and a relatively low number of dropouts. The most outstanding toxicity reactions are asthenia, neuropsychiatric symptoms, myelosuppression, alteration of liver enzymes, and so forth. The neuropsychiatric effects may appear early or tardive and include signs of depression, anxiety, and occasionally suicidal thoughts (Tables 9 and 10) [37–39].

In conclusion, there are arguments in favour of the use of high dose interferon-*α*2b as this treatment shows improvement of disease-free survival in all studies carried out to date and increased, but not statistically significant, global survival. The toxicity is undoubtedly high, but manageable in services with experience. Furthermore, there is no other therapeutic regime that has shown benefits in adjuvant treatment of melanoma. However, there are also arguments against high dose interferon-*α*2b. In the first place it is not clear which patient population really benefits from adjuvant treatment. The only clear benefit is for disease-free survival; no consistent data is available for global survival. In second place the toxicity is considerable and requires a team with experience in its management even though a certain number of patients will abandon or suspend treatment for this reason. Finally the duration and ideal dosage for the treatment is unknown (Table 11) [37].

Other favourable arguments are the data from a study that analyzed the quality adjusted survival (QAS) using clinical data from the E1684 and E1690 studies which pointed out that the majority of patients showed improvement of the QAS, but the benefit was only significant in 16% of patients in the E1684 study [40]. 

A cost-effectiveness analysis of high dose interferon-*α*2b as adjuvant treatment for high risk melanomas in Spain shows that it is within established limits for healthcare economy regarding the use of a new treatment [41]. Another recent study of cost effectiveness in node positive melanomas shows that the treatment was cost effective, even though it varied according to the substage, and also highly effective in terms of quality of life per year in patients under 60 years of age with stage IIIC melanoma [42].

Even more recently a stage III study was published comparing i.v. induction of interferon *α*2b to the classic high dose scheme with induction and maintenance. At 51 months of followup, the RFS was 32 months versus 31 months (*P* = 0.836) and the OS was 61 months versus 63 months (*P* = 0.444), without significant differences. There were more dropouts in the classic treatment (*P* < 0.001), mainly because of its duration and signs of recurrence being more than for toxicity. This study, which included 355 patients, attempted to show the value of induction (no differences between both groups), but lacked an untreated control group to confirm this in a more evident way. However, this group was not considered after the published data reported on the benefits of adjuvant treatment [43].

Another similar study presented in ASCO 2010 showed that patients in stages IIB and IIIA have similar RFS and OS in both groups, the ones with induction plus 8 weeks of maintenance dose and the ones with high doses according to Kirkwood regime [44]. In high risk melanomas there is another study in phase III with 364 patients that compares 4 weeks of induction versus 1 year of treatment with classic high doses of IFN, showing no significant differences in RFS and OS between both regimes [45]. There is also another phase III randomized study from DeCIG MM-ADJ-5 with 380 patients in stage III that compares 3 treatments with IFN *α*2b 20 MU/m^2^ i.v, five days a week for four weeks every four months and the classic regime of high doses of IFN from Kirkwood, showing no significant differences in DMFS, but with better tolerance and safety with the intermittent treatment [46]. Therefore shorter regimes might encourage the use of IFN as an adjuvant treatment in melanoma patients.

As a final summary it can be said that in patients with high risk resected melanoma, high dose interferon is the adjuvant treatment to be proposed, together with background information on its collateral effects, as there is clear and significant evidence of improvement in the RFS and moderate, although not significant, improvement of the GS.

New data have recently been published on high dose interferon-*α*2b according to the Kirkwood scheme as neoadjuvant treatment prior to lymphadenectomy, in patients with palpable stages IIIB and IIIC adenopathies. After four weeks of intravenous phase among the 20 patients enrolled, 11 (55%) showed response, three of them (15%) pathological complete response. At a median of 18.5 months followup, 10 patients continued disease-free. In responding patients the cells CD11+ and CD3+ increased to the level of the tumour and CD83+ decreased, indicating a correlation between reactivity of the immune system and the benefit of the treatment. This study also included molecular analysis with activation of STAT3 being observed and related to cell proliferation; high dose interferon-*α*2b would reduce this protein and increase STAT1 This enables opening a new approach to adjuvant treatment in high risk patients which should be more widely explored [52].

Low and intermediate doses have not shown any real benefits in the adjuvant treatment of high risk melanomas (Table 12) [53]. But in the review by Verma and col. [54] with patients with high risk melanomas, the results show that the treatment with high doses of IFN constantly improve the SLR and the mortality rate at two years (*P* < 0.03). The authors conclusion is that IFN at high doses is a reasonable option in selected patients. A recent meta-analysis evaluating 6067 patients from 10 trials found significant benefits in RFS and GS (*P* = 0.00006 and *P* = 0.008), even though the absolute benefits on survival are small, just a 3% at five years. This meta-analysis did not clarify the ideal dose of interferon nor the duration of the treatment and found a subgroup where the benefits were greater, in the presence of ulceration in the primary tumour, but this needs clarification [55].

A recent phase III randomized study from DeCOG has compared low doses of IFN *α*2b (3 MU three times a week) for 18 months (group A) versus 60 months (group B), in patients with primary melanoma, a Breslow's thickness ≥1.5 mm, and negative lymphadenopathies clinically. 75.6% of them had a sentinel lymph node biopsy, with a positive results in 18% in group A and 17.5% in group B. Overall they had 840 patients, with a median followup of 4.3 years, and it did not show any benefits with prolonged treatments. All this suggests that the optimal length of the treatment with IFN is still nuclear [56].

**Figure 4:**
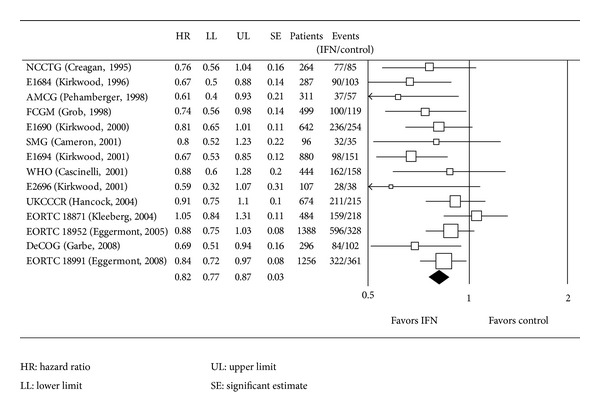
Meta-analysis. Disease-free survival [61].

**Figure 5:**
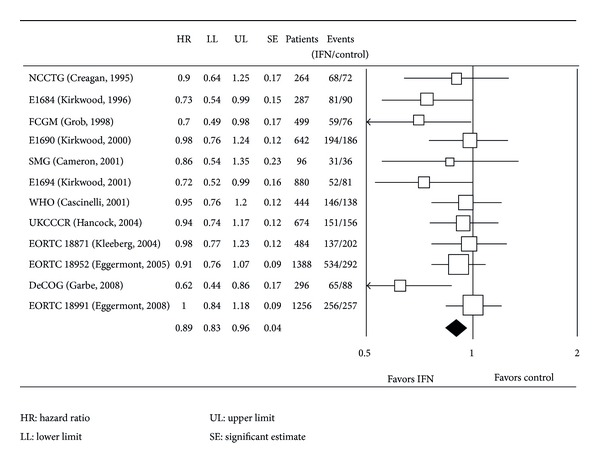
Meta-analysis. Overall survival [61].

Some data on adjuvant treatment with pegylated interferon *α*2b (PEG-IFN) has been published recently from the EORTC 18991 study (induction of 6 *μ*g/Kg/week, s.c. for 8 weeks, followed by maintenance at the dose of 3 *μ*g/Kg/week, s.c., for a total duration of 5 years). The study included 1256 patients in stage III (any T, N1-2, Mo, without metastasis in transit). The patients were randomized into two groups, one for treatment (608 p.) and the other for observation as a control (613 p.). The randomization was divided into positive microscopic lymphadenopathy (N1) versus macroscopic one (N2), number of positive lymph nodes, tumour ulceration and Breslow's thickness, sex of the patient, and the referral center, analyzing the data according to the intention of treatment. The average length of treatment with PEG-IFN was 12 months (IQR: 3.8–33.4). The mean followup was 3.8 years, and 328 recurrences were observed in the interferon group and 368 in the control one (*P* = 0.01), with the RFS value being 45.6% in the first group and 38.9% in the second one at four years, showing a risk reduction of 18% (*P* = 0.01). No significant differences were observed between the two groups in OS. Grade 3 adverse event occurred in 246 (40%) patients in the interferon group and 80 (10%) in the observation group; grade 4 adverse events occurred in 32 (5%) patients in interferon group and 14 (2%) in the observation group. In the interferon group the most common grade 3/4 adverse events were fatigue (97%/16%), hepatotoxicity (66%/11%) and depression (39%/6%). Treatment with PEG-IFN was discontinued because of toxicity in 191 (31%) patients. Regarding the quality of life, there was a negative effect in the group treated with IFN with a decrease in social activity and appetite. Knowing that PEG-IFN increases the RFS, the patients should be informed of the negative effects of the treatment and they should be encouraged to participate in the planning of the treatment (Table 13) [57–59]. The EADO trial is a phase III study with excised melanomas ≥1.5 mm of thickness and with no affected lymph nodes clinically; the patients were randomized to receive IFN*α*-2b (3 MU subcutaneously three times a week for 18 months) versus PEG-IFN (100 mcg, subcutaneously once a week for 36 months). Out of 898 patients included, 896 were evaluated (453 IFN and 443 PEG) with a mean followup of 4.7 years. Sentinel node biopsy was performed in 68.2% because it was not a standard procedure initially. The recurrence-free survival (RFS) was 64.8% versus 66.2% (*P* = 0.43), the distant metastasis-free survival (DMFS) was 72.6% versus 71.3% (*P* = 0.55), not showing significant differences. Adverse effect of grades 3-4 were seen in 26.6% versus 44.6% in the first 18 months, which affected the mead length of treatment (17.8 months versus 19.2 months, completing the full 36 months of treatment 28% of the patients). In summary, low doses of PEG-IFN were no better than low doses of conventional IFN*α*-2b. Trying to increase the benefits of PG-IFN by increasing the length of the treatment to three years is not easy because of the high numbers of patients not completing the full treatment due to the side effects and therefore not solving the clinical needs of them [60]. Advocating the use of IFN in melanomas, a new meta-analysis has recently been published with a large number of patients reviewing the adjuvant treatment with IFN-*α* in high risk cases, in relation to DFS and OS, and also the effect of the doses and the length of the treatment has been studied. 14 randomized studies have been included between 1990 and 2008, with a total of 8122 patients, out of which 4362 were treated only with IFN-*α* and the rest were only observed. The treatment with IFN-*α* is associated to the improvement of the DFS (*P* < 0.001/18% risk reduction) and also of the SG (*P* = 0.002/11% risk reduction) (Figures 4 and 5). The study has its own limitations according to the authors and therefore it cannot recommend the regime, doses, or length of the treatment nor which subgroup of patients would respond better to the adjuvant therapy. Given the lack of an effective systemic treatment to treat the melanoma, the meta-analysis suggests the use of IFN-*α* on the daily clinical bases to offer the patients the best survival opportunities. It is important to remember that other adjuvant therapies well established for other types of cancer like breast, colorectal, and ovarian are associated with a risk reduction. These data suggest that it is very important to research the molecular mechanism that could explain the sensibility to the IFN-*α* to try to identify the group of patients that would benefit most from it [61].

Mucous membranes melanoma is an entity that must be considered independently in relation to adjuvant treatment. A study presented in the ASCO 2012 meeting [62] compared observation (A) with high dose IFN in standard regimen (B) and chemotherapy (Temozolomide + cisplatin) (C) every three weeks for six cycles, after surgery of primary mucosa melanoma with a total of 184 evaluable patients. After a followup of 26,8 months, SLR was 5.4, 9.4, and 20.8 months, respectively (*P* < 0.001). The OS was 21.2, 41.1, and 49.6 months (*P* < 0.001), with toxicity being generally moderate. In conclusion, this study shows that chemotherapy improves significantly RFS and OS, so this subset of melanomas should be considered as a different entity due to its greater agressivity.

Given the characteristics of adjuvant treatment with interferon-*α*2b, it would be extremely important to find factors predicting efficiency and parameters for classification of patients to enable a better choice of therapy. Autoimmunity seems to be a factor predicting efficiency in adjuvant treatment with interferon. A prospective study with high dose interferon analyzed the autoimmune response through the appearance of thyroid, anti-cardiolipins, anti-nuclear, and anti-DNA autoantibodies or the presence of depigmentation. A quarter of all patients treated develop autoimmunity phenomena. After a followup of 45.6 months, only 13% of those presenting autoimmunity had suffered relapse and 4% had died. In the group that presented no autoimmunity reactions, 73% suffered relapse and 54% died. The mean survival has not been reached among the patients with autoimmunity phenomena and was 37.6 months in the group without these manifestations. Therefore, after multivariate analysis, autoimmunity constitutes a significant predictive factor for global and disease-free survival [63]. 

On the other hand, the trial 18991 from EORTC where adjuvant IFN was compared versus control, the presence or not of autoantibodies (anti-cardiolipin, anti-thyroglobulin, and anti-nuclear) did not represent an important prognostic factor and did not find a significant relationship with the treatment [64].

The determination of HLA is also a factor predicting recurrence in patients treated with interferon *α*2b as adjuvant treatment. The percentage of relapses is significantly lower in patients with HLA genotype A33, HLA B57, HLA-Cw03, and HLA-Cw06 [65].

It therefore seems essential to be able to discriminate those patients who would really benefit from adjuvant treatment with interferon *α*2b, thus avoiding all side effects in patients that would not really benefit. In addition, this would also have a considerable economic impact.

### 2.6. Adjuvant Treatment with GM-CSF

The GM-CSF is an important hematopoietic growth factor, codified by a gene placed in the long arm of chromosome 5 (5q21–q32), present in monocytes, fibroblasts, and endothelial cells, with a stimulating action over the developing and maturation of stem cells that will become neutrophils, eosinophils monocytes, and macrophages. It has been used therapeutically to treat QT induced neutropenias. The in vivo studies have shown that recombinant GM-CSF increases the cytotoxic activity of monocytes and lymphocytes and also increases the activity of macrophages by increasing the production of matrix metalloproteinases and angiogenesis inhibitors, and showing therefore greater antitumoral effect, together with the increased immunogenicity of the tumoral cell, facilitating the antigen presentation. The reason for its use as an adjuvant therapy in excised high risk melanoma is because it also induces dentritic cell differentiation.

In 2000 the first results were published on GM-CSF showing benefits on survival in relation to historic controls in stage III patients with a poor prognosis or stage IV with resected disease. Recent data on 98 high risk patients under treatment for three years show a mean survival of 58.7 months, longer than the result of 37.5 months obtained in the first study where treatment only lasted one year. The benefits were especially observed in stage IIIc. The conclusion was the superiority of long-term treatment over three years, especially in patients that maintained eosinophilia for a longer period of time [66]. 

This study has been reviewed recently and once again they conclude that GM-CSF for three years increases the survival in patients with a high recurrent risk of melanoma (HR = 0.61; *P* = 0.047), but those three years of treatment have the side effect of potentially causing *AML,* as happened in two patients. Immunological studies showed an increase of neopterin related to the macrophages activity, that potentially could explain the mechanism of action of the therapy [67]. The phase III study E4697 that compares GM-CSF versus placebo as an adjuvant treatment in staging III-IV melanoma that were excised, included 815 patients (1999 to 2006), out of which 735 were eligible. The overall mean survival rate was 72.1 versus 59.8 months (*P* = 0.551) and the disease-free survival was 11.8 versus 8.8 months (*P*-0.034), with a minimum toxicity [68]. Undoubtedly the use of GM-CSF as an adjuvant treatment either as monoterapia or in combination is a research pathway that must be confirmed over the next few years.

### 2.7. Adjuvant Radiotherapy

It is an option in melanomas with a high risk of regional recurrence after lymphatic clearance, specially in those cases with extra lymphatic extension, a positive lymph node greater than 3 cm, 4 or more positive nodes, residual disease, or a Breslow's thickness equal to or greater than 4 mm. In a randomized study with 227 patients, considered as having a high risk of recurrence, 109 were included in the adjuvant radiotherapy group and 108 were considered control. After a mean followup of 27 months, 20 patients had recurrences in the radiotherapy group and 34 in the control (*P* = 0.0410), indicating a better control of the local recurrences with radiotherapy but not affecting the survival rate [69].

### 2.8. New Treatments

The lack of proven efficient treatments against metastatic melanoma affects the use of the adjuvant treatment. Chemotherapy, cytokines, vaccines, and combination of the treatments have been studies with little success. Only IFN has shown to be beneficial in DFS and in less degree in OS in high risk patients, therefore it is necessary to continue to research for new therapies. A new line of research has been found in the monoclonal autoantibodies anti-CTLA-4 that block the interaction between B7 (B7-1 and B7-2 are homologous costimulatory ligands expressed on the surface of antigen presenting cells) and CTLA-4 (cytotoxic T-lymphocyte antigen 4), causing a negative inhibition that increases the cytotoxicity of T-lymphocytes with antitumoral activity [70]. At present, there are two monoclonal antibodies on phase II/III trials, used on their own or in combination: Ipilimumab and tremelimumab.

Early-phase (I/II) clinical studies of tremelimumab demonstrated acceptable toxicity, mostly immune-related adverse events and similar efficacy of 10 mg/kg monthly and 15 mg/kg quarterly doses of the antibody with median survival times of 10.3 and 11 months, respectively. Both phase II regimens generated durable tumor response [71]. Interesting data were presented at the 2010 annual meeting of the ASCO (American Society of Clinical Oncology), regarding the combination of tremelimumab and HDI (high dose interferon alpha-2b) in a phase II trial in patients with metastatic melanoma. With an overall response rate of 30%, a progression-free survival rate at 6 months of 53%, and a median OS of 15.9 months, the results are very encouraging, especially since there seemed to be no added toxicity associated with the combination of tremelimumab and HDI. Autoimmunity induced by therapy is significantly correlated with therapeutic benefit [72].

There are several studies in the last few years about metastatic melanoma and Ipilimumab as the only treatment and in combination with DTIC with survival rates of 11.5 and 13 months respectively. The presence of autoimmune reactions (diarrhoea, colitis and dermatitis) that can be controlled with steroids, can be used as a marker to asses the respond and duration of the treatment. A phase III study compares ipilimumab and placebo (137 patients) versus ipilimumab and vaccine gp100 (403 patients) versus placebo and vaccine gp100 (136 patients) in unresected stage III or stage IV melanoma previosly treated. The main aim of this study was the overall survival rate (OS). The mean survival was 10.0 months for the group that had Ipilimumab and gp100 and 6.4 months for the group that had gp100 alone (*P* = 0.001). The survival of the patients who only had Ipilimumab was 10.1, also better than the gp100 alone group (*P* = 0.003), not showing any differences between the two groups that had Ipilimumab. Immune reactions of grades 3-4 were seen in about 10–155 of patients treated with Ipilimumab and 7 deaths were related to these side effects. In summary, Ipilimumab is the first drug that increases the survival in patients with advanced melanoma previously treated [73]. The DTIC-Ipilimumab association achieves an increase of OS related to DTIC in patients with stage III unresected and stage IV previously untreated. A systematic review based on “randomized” studies indicates that the median survival is greater with Ipilimumab plus DTIC related to other combinations in patients with stage III unresected/IV previously untreated (Table 14) [74]. A study presented in the ASCO 2012 meeting [75] shows the results of the association of Ipilimumab plus DTIC versus DTIC plus placebo, being two-year survival (28% versus 18%) and three-year survival (20.8% versus 12.2%) (*P* < 0.001), with a good safety profile. There are only preliminary data of the combination of Ipilimumab plus Temozolomide or Fotemustine, but more extensive research is required [76, 77].

The lack of benefit observed in stage IIIB/C with adjuvant IFN therapy was, for the EORTC Melanoma Group, the reason to move to a different drug. Thus, the EORT C 18071 pivotal adjuvant trial in stage IIIB/C, comparing a 3-year treatment with Ipilimumab to placebo in a double-blind randomized setting, was activated in 2009 and is expected to complete accrual in 2011.

Vemurafenib is an inhibitor of the BRAF V600 mutation that has recently been approved by the FDA for metastatic melanoma treatment in adults, in the absence of brain metastases, with a response rates around 50% on published data. In a phase II study with 132 patients with previously treated metastatic melanoma, vemurafenib achieves a 53% objective response (CR: 6% and PR: 47%). The median duration of response was 6.7 months with a DFS average of 6.8 months and with a median OS of 15.9 months. The most common adverse events were arthralgia, rash, photosensitivity, fatigue, and alopecia (grades 1 and 2) [78]. In a study with 675 patients with metastatic melanoma and BRAF V600 mutation, previously untreated, patients were randomized to receive vemurafenib (960 mg twice daily orally) or DTIC (1000 mg/m^2^ every 3 weeks). Six months OS was 84% versus 64% with an improvement of the DFS (*P* < 0.001). The overall response rate was 48% versus 5%, with the usual toxicity study drug [79].

MEK trametinib, one mutated BRAF selective inhibitor, has been investigated in a phase III trial, where 322 patients were enrolled with metastatic melanoma with mutated BRAF V600E or V600K. Patients were randomized 2 : 1 to receive trametinib (2 mg. once daily orally) or DTIC (1000 mg/m^2^) or paclitaxel (175 mg/m^2^) every three weeks. Patients who progressed to chemotherapy were allowed to receive trametinib. The conclusion was that trametinib improved PFS and OS in patients with BRAF V600E mutation or V600K [80]. Other drugs are currently under investigation in combination with chemotherapy such as Lenvatinib, Pazopanib, Dabrafenib, Axitinib, Everolimus, Bevacizumab, and so forth [81, 82].

## 3. Followup

Followup aims to identify asymptomatic metastases and locoregional recurrence. In a study of 261 patients with stages II-III followed prospectively, symptoms of melanoma recurrence were announced in 99 of 145 patients who relapsed (68%). Physical examination detected asymptomatic recurrence in 37 patients (26%). Chest radiography detected only 9 of 145 recurrences (6%) and no recurrence was found in any laboratory tests. Medical history and physical examination detected 94% of recurrences, hence their importance, which was later confirmed by other tests [83]. Another study of 1004 patients with stages I-II followed by clinical history, physical examination, laboratory tests, and chest radiography detected 72% of recurrences by physical examination, 17% by symptoms, and 11% by chest radiology [84].

Therefore, clinical history and physical examination are the bases for monitoring patients with localized disease followed by chest X-ray. Other complementary studies are less relevant due to their lower diagnostic yield (Table 15).

## 4. Final Comments

The reality is that except for data on IFN, no new validated strategies that improve results have come to the fore. The unquestionable increase of our understanding of the cell biology of melanomas leads to the idea of identification of subgroups where the benefits would be greater. It is therefore absolutely necessary to identify new therapeutic targets, develop new drugs, and make an optimal selection of patients. One of the most interesting targets is analysis of the BRAF gene, mutated in 50–70% of melanomas, and furthermore associated with exposure to ultraviolet light. This mutation gives rise to a protein with a kinase activity about 500 times higher than the unmutated protein thus enabling greater survival and proliferation of neoplastic cells. Sorafenib, a double target antiangiogenic which inhibits BRAF on the one hand and VEGFR and PDGFR on the other hand, in association with CDDP in metastatic melanomas results in 13% PR and 53% SD. PD0325901 is another important inhibitor of the BRAF signal cascade (MEK1 and MEK2) and its efficiency has been tested in preclinical models as well as PLX40323 [85].

Vemurafenib and trametinib, mutated BRAF inhibitors, may play an important role in adjuvant treatment, associated or not to chemotherapy/interferon. The CTLA-4 inhibitors as Ipilimumab and tremelimumab also open a new era in the treatment of melanoma, and adjuvant studies may change the outcome, especially in those at high risk. In the future we will have to analyze all these therapeutic possibilities on specific targets, probably associated with chemotherapy and/or interferon in the adjuvant treatment if we want to change the natural history of melanomas.

## Figures and Tables

**Table 1:** Histological criteria indicating the sentinel lymph node.

(1) Tumour thickness of more than 1 mm
(2) Clark level higher than III
(3) Ulceration
(4) Histological signs of regression

**Table 2:** Prognostic factors in stage III.

Factor	Value of “*P*”
Patient age	<0.0001
Male versus female	0.12
Primary location	0.002
Ulceration of primary tumour	0.13
Breslow thickness	0.05
Number of positive nodes	<0.0001
Clinical affectation of nodes	0.0003
Micro- versus macrometastasis in the lymphatic nodes	<0.001
Extranodal extension	0.07.

**Table 3:** Melanomas: risk groups.

(1) Low risk melanomas	
Stage I	
(2) Intermediate risk melanomas	
Stage II A	
(Breslow of 1.1–2 mm ulcerated)	
(Breslow of 2.1–4 mm nonulcerated)	
(3) High risk melanomas	
Stage IIB	
(Breslow of 2.1–4 mm ulcerated)	
(Breslow > 4 mm nonulcerated)	
Stage IIC	
(Breslow > 4 mm ulcerated)	
Stage III	

**Table 4:** Molecular subgroups according to locations melanomas.

Arising from skin without chronic sun damage	50% BRAF 20% NRAS	0% KIT
Arising from skin with chronic sun damage	10% BRAF 10% NRAS	2% KIT
Arising from mucosal surfaces	5% BRAF 15% NRAS	20% KIT
Arising from acral surfaces	15% BRAF 15% NRAS	15% KIT
Uveal melanoma	25% GNAQ	55% GNA11

**Table 5:** Melanomas: adjuvant chemotherapy. Randomised studies [20].

Authors	No. of cases	Stage	Treatment	Followup (years)	Statistical significance
Fisher 1981	181	II-III	CCNU	3 y.	NS
			Observation		
Veronesi 1982	931	II-III	DTIC	5 y.	NS
			BCG		
			DTIC + BCG		
			Observation		
Lejeune 1988	325	I-IIA-IIB	DTIC	4 y.	NS
			Levamisole		
			Placebo		
Meisenberg 1993	39	III	Autologous bone marrow transplant	NA	NS

NA: not announced. NS: not significant.

**Table 6:** Melanomas. Adjuvant treatment with nonspecific immune stimulants. Randomised studies [20].

Authors	No. of cases	Stage	Treatment	Followup (years)	Statistical significance
Balch 1982	260	III	*C. parvum *	2 y.	NS
Paterson 1984	199	I-II	BCG	4 y.	NS
Miller 1988	168	II-III	Transfer factor	2 y.	NS
			Observation		
Lipton 1991	262		*C. parvum*	4–9 y.	CS
			BCG		
Quirt 1991	577	I-IIA-IIB	Levamisole		
			BCG		
			BCG + Levamisole		
			Observation		
Spitler 1991	216	I-IIA-IIB-III-IV	Levamisole	10 y.	NS
			Observation		
Czarnetzki 1993	353	II	BCG (RIV)	6 y.	NS
			BCG (Pasteur)		
			Observation		

CS: close to significance. NS: not significant.

**Table 7:** Melanomas. Adjuvant treatment with vaccines. Randomised studies [20].

Authors	No. of cases	Stage	Treatment	Followup (years)	Statistical significance
Livingston 1994	123	III	GM2 + BCG + CFM	5 y.	NS
			BCG + CFM		
Wallack 1995	250	II	Virus allogeneic polyvalent melanoma cell lysate	2.5 y.	NS
Wallack 1998	250	III	Melanoma cell lysate vaccine	3 y.	NS
Bystryn 2001	38	III	Polyvalent shed antigen	2.5 y.	S
			Placebo		
Sondak 2002	689	IIA	Melacine and DETOX	5.6 y.	NS
			Observation		
Hershey	700	IIB	Cell lysate vaccine	8 y.	Tendency in RFS/GS
			Placebo		

**Table 8:** Final results of study EORTC 18961 [26].

	RFS		DMFS		OS	
	OBS	GM2-KLH/QS-21	OBS	GM2-KLH/QS-21	OBS	GM2-KLH/QS-21
No. of events	204	205	143	152	112	124
4 yr% (SE%)	69.4% (1.9%)	68.2% (1.9%)	78.8% (1.7%)	76.1% (1.8%)	83.6% (1.6%)	81.5% (1.6%)
HR (95% CI)*	1.03 (0.84, 1.25)		1.11 (0.88, 1.40)		1.16 (0.90, 1.51)	
*P* value*	0.81		0.36		0.26	

HR: hazard ratio. *Cox model: stratified for stratification factors. OBS: observation. RFS: relapse-free survival. DMFS: distant metastasis-free survival.

OS: overall survival.

**Table 9:** Indications, cautions, and contraindications for adjuvant treatment with high dose interferon.

Indications:
(i) High risk melanomas
(ii) Patients with ECOG 0-1
Cautions on:
(i) Diabetes mellitus
(ii) Cardiovascular disease
(iii) Chronic lung disease
(iv) Chronic kidney disease
Contraindications:
(i) Pregnancy and lactation
(ii) Children
(iii) Autoimmune diseases
(iv) Immunosuppression (organ transplanted)
(v) Decompensated liver disease
(vi) Neuropsychiatric disease (depression)
(vii) Myelosuppression
(viii) Infections on treatment

**Table 10:** Most common adverse events (degree III/IV) in patients treated with high dose IFN-*α*2b.

	Patients (%)*	Patients (%)*
Adverse effects	All degrees	Degree 3-4
Asthenia	96	21–24
Fever	81	18
Myalgia	754	4–17
Nauseas	66	5–9
Vomiting	66	5
Myelosuppression	92	26–60
Elevated AST	63	14–29
Neuropsychiatric symptoms	40	2–10
(i) Depression		
(ii) Anxiety	0–70%	
(iii) Suicidal thoughts		

*Data taken from the E1684 study of 143 patients. **Data taken from the E1684, E1690, and E1694 studies.

**Table 11:** Arguments for and against high dose interferon as adjuvant treatment.

(1) Arguments in favour:
(i) A consistent improvement in disease-free survival has been demonstrated in all studies carried out
(ii) An improvement in global survival has been shown, but without this being statistically significant
(iii) The toxicity is high, but manageable by experienced medical teams
(iv) No other therapeutic regime has shown benefits in the adjuvance of melanoma

(2) Arguments against:
(i) It is not clear which population most benefits from the adjuvant treatment
(ii) The benefit is only clear for disease-free survival, with no consistent information referring to global survival
(iii) The toxicity is considerable
(iv) The ideal duration and dose for the treatment are unknown

**Table 12:** High risk melanomas: adjuvant treatment with low and intermediate dose interferon.

Trial	No. of cases	SLE	OS
Low dose IFN (3 mU × 3/s × 3 y.):			
WHO-16 (Cascinelli et al.2001) [47]	426	NS	NS
UK [48]	674	NS	NS
Scottish study (2001) [49]**	59	NS	NS
Ultralow dose IFN (1 mU):			
EORTC/DKG-80 (Eggermont. 2001) [50]	830	NS	NS
Low dose INF + Isotretinoin (IFN: 3 mU × 3/s × 2 y.)			
ECAMTSG ([51])	407	NS	NS
Intermediate dose IFN.			
EORTC 18952*	1388	NS	NS
(10 mill. 13 m versus 5 mill. 25 m. versus observation)			

Eggermont et al. Semin. Oncol. Spanish Ed. 3, 221–227, 2003*.

Hancock et al. J. Clin. Oncol. 22, 53–61, 2004 [48].

Cameron et al. BJC. 84(9): 1146–1149, 2001**

Richtig et al. JCO. 23, 34, 2005 [51].

**Table 13:** Pegylated interferon. Results of the EORTC 18991 study [57].

	RFS		DMFS		OS	
	Obs.	PEG-IFN	Obs.	PEG-IFN	Obs.	PEG-IFN
No. of events	368	328	325	304	263	262
Rates at 4 years	39%	46%	45%	48%	56%	57%
Mean years	2.1	2.9	3.0	3.8	NR	NR
HR (95% CI)	0.82 (0.71–0.96)		0.88 (0.75–1.03)		0.98 (0.82–1.16)	
Value of “*P*”	**0.01**		0.11		0.78	

**Table 14:** Systematic data review.

SP (%; 95% credible interval)						
	Month 6	Month 12	Month 24	Month 36	Month 48	Median OS
DTIC	60.9 (58.5–63.2)	36.3 (33.6–39.2)	12.1 (9.3–15.4)	3.7 (01.09–06.03)	1.0 (0.2–2.7)	11.0 (10–12.2)
DTIC + IFN	60.9 (53.9–67.2)	36.9 (29.6–44.5)	13.5 (7.3–21.7)	4.9 (1–12.7)	NA	11.5 (9.2–15.4)
DTIC non IFN	59.7 (50.3–67)	35.6 (26.4–43.9)	12.7 (6.8–20)	4.6 (1.1–11)	1.7 (0.1–7)	11.2 (8.7–14.5)
DTIC + Oblimersen	64.2 (58.4–70.5)	41.2 (34.9–48)	16.8 (11.3–23.2)	NA	NA	12.9 (10.8–16)
TMZ	64.8 (60.1–69.5)	39.5 (34.2–44.9)	11.9 (7.3–17.5)	2.5 (0.5–6.8)	NA	11.3 (9.8–13.2)
TMZ + IFN	67.6 (59.4–75.2)	43.6 (34.4–53.3)	15.2 (8–24.5)	4.0 (0.6–11.9)	NA	12.5 (10.1–16.1)
TMZ non IFN	69.6 (61.1–78.4)	45.5 (34.9–56.8)	15.0 (6.4–27)	NA	NA	12.6 (9.9–17.1)
DTIC + IPI	**70.2 (63.8–75.3)**	**48.4 (40.7–55.1)**	**21.3 (14.8–28.7)**	**8.5 (3.6–15.6)**	**3.1 (0.5–9.2)**	**14.7 (12.2–18.1)**

**Table 15:** Melanoma. Followup.

Stage	1st year	2nd year	3rd, 4th, 5th years and beyond
Stage I			
Medical history and physical examination	Every 6 months	Every 12 months	Every 12 months
Stage II			
Medical history and physical examination	Every 3 months	Every 6 months	Every 12 months
Blood test	Every 6 months	Every 12 months	Every 12 months
Chest X-ray	Every 6 months	Every 12 months	Every 12 months
Abdominal ultrasound	Every 12 months	Every 12 months	Every 12 months
Stage III			
Medical history and physical examination	Every 3 months	Every 6 months	Every 12 months
Blood test	Every 6 months	Every 12 months	Every 12 months
Chest X-ray	Every 6 months	Every 12 months	Every 12 months
Abdominal ultrasound	Every 12 months	Every 12 months	Every 12 months

Optional			
Brain CT scan, thoracoabdominal CT scan, bone scan, and/or FDG-PET as clinical assessment			

Stage IV			
Additional tests depending on the patient's clinical features and treatment performed			
